# Identification of Potential or Preclinical Cognitive Impairment and the Implications of Sophisticated Screening with Biomarkers and Cognitive Testing: Does It Really Matter?

**DOI:** 10.1155/2013/976130

**Published:** 2013-07-08

**Authors:** Michael Gordon

**Affiliations:** Baycrest Centre for Geriatric Care, University of Toronto, 3560 Bathurst Street, Toronto, ON, Canada M6A 2E1

## Abstract

The last decade has seen an enormous growth in the interest in the recognition of and intervention in those diagnosed and living with the whole range of cognitive impairment and frank dementia. In the western world, the recognition of the impact on patients, families, health care systems, and societies that dementia poses has led to great efforts to help define the indicators for current and future dementia with the intention to treat those already afflicted even with the primarily symptomatic medications that exist and to recognize those at future risk with the hope of providing counselling to forestall its future development. The idea of “early diagnosis” appears at first glance to be attractive for the purposes of future planning and research studies, but it is not clear what the benefits and risks might be if screening processes define people at risk when beneficial interventions might not yet be determined. The ethical as well as financial implications must be explored and defined before implementation of such screening becomes a normal standard of practice.

## 1. Introduction

One of the usual dictums of modern medicine is the concept of “early diagnosis.” Whether it is screening for metabolic disorders at birth, hearing deficiency, and eye infections, orthopaedic dysfunction processes to routinely screen for such problems has become part of the standard of care to avoid, prevent, or mitigate significant illness, disability, or even death later on. During adult years, we have become imbued with the accepted concept that screening for colon, cervical, and breast cancer, thyroid deficiency, lipid and glucose abnormalities, and hypertension have all been shown to have some measurable benefit for the person in question of future health and well-being. We have also learned to be somewhat chastened by the enthusiasm for screening for some conditions as we have seen in the prostate screening and treatment controversy. What was previously a concerted “hunt” for elevated PSA levels has been subdued because of the unanticipated negative effects that screening and subsequent treatments conferred on individuals with much less benefit than anticipated [1].

## 2. Early Diagnosis and Cognitive Impairment

 One of the very controversial questions that exist at present in the professional community is whether or not there is any merit to “early diagnosis” of minimal and not clinically relevant cognitive impairment. This might be revealed through neuropsychological testing and identification of those individuals at apparent high risk for future cognitive impairment or dementia. This would presumably be based on a battery of tests which might include sophisticated imaging techniques and/or biomarkers that have been shown to be associated with higher risk factors for disease. There is a growing body of knowledge about whether or not one might improve the predictability of future dementia development as well as testing that might reveal subtle cognitive changes that also might predict future clinically significant cognitive decline. A pressing question is what, if any, is the merit of using these modalities of screening and testing on the “normal” or “potentially at risk” population as well as the so-called worried well who may seek assurance that they are not at risk. This is especially the case for family members of those already living with or previously experiencing or dying from dementia as part of their quest to decrease their own risk, at times in an understandably fearful or even obsessive manner.

 A recently published on-line Associated Press article posted on March 19, 2013, revealed that 1 in 3 seniors die with dementia in the US. Even when dementia is not the direct cause of death, it can speed someone's decline [2]. As the article notes, “Dying *with *Alzheimer's is not the same as dying from it. But even when dementia is not the direct cause of death, it can be the final blow—speeding someone's decline by interfering with their care for heart disease, cancer or other serious illnesses.” The report released on March 19, 2013, by the Alzheimer's Association noted that “already, 5.2 million Americans have Alzheimer's or some other form of dementia. Those numbers will jump to 13.8 million by 2050. That's slightly lower than some previous estimates” [3, 4].

 Such figures imply many things including the potential increase in demands and costs to the health care system, [5, 6] the increased responsibility with associated emotional and financial costs to families, and the quest on the part of the health care and pharmaceutical industries to develop products that may change the trajectory of the condition. The ultimate goal would be to eliminate the condition or at least ease the processes of care carried out at the micro- and macrolevels. The “dementia industry” is huge with many players including the individual patient and family support group; those that manufacture pharmaceuticals; those that do the research; those that run the dementia-based institutional networks that have sprouted all over the place, all those health care and social service professionals that are involved in front-line care. In addition, there are the policy makers and their supports that try and hammer out “solutions” that benefit the public and the public/private purse [5, 6].

 When one examines some of the recent initiatives in the dementia industry, the quest for early diagnosis has become a major theme. Some of this thrust has come within the past few years when various so-called *biomarkers* have been discovered that appear to have a close correlation with the likelihood of developing dementia. Some genetic findings also seem to point in the same direction but as of yet there is no one test that predicts with certainty whether or not a person having the test in their mid or later-mid years is absolutely certain to develop a dementia of Alzheimer's disease or other type in the future. Because of this uncertainty in predictions and the implications of receiving a “predisease” diagnosis, an editorial written by Gauthier et al. in Alzheimer's and Dementia in 2011 emphasized the potential ethical and financial risks to society and the individual if some of the recommendations that had been promoted and commented upon in the editorial are implemented [7].

 In the reports of the Canadian Consensus Conference on the Diagnosis and Treatment of Dementia 4, similar recommendations were made about being very cautious before entering the very lucrative diagnostic “preclinical” fray as the personal and societal implications are profound. Moreover, in the absence of what would be defined as effective pharmacological therapy, it is hard to justify the benefits of such testing other than for the primary purposes of research [8].

 When one addresses the issue of being *at risk* for developing cognitive impairment/dementia, the question that is often raised is how far into the future are we talking about? There is good evidence that the prodromal period prior to clinical evidence of cognitive impairment can be many years and even with those with a clinical determination of mild cognitive impairment can have a very variable conversion to dementia with some evidence that various combinations of neuropsychological testing and other markers might be more predictive of conversion [9–13]. A question that is worth asking is whether it is crucial in the care plan of the individual to be better at predicting the *likelihood* of development of dementia at some time in the future. Will such a prediction change actions and attitudes in that person and his loved ones in a positive manner and potentially useful manner? 

 If such a determination might promote planning for the future in terms of such things as wills, advance directives, estate planning, and potentially beneficial life-style changes, it might be possible to support such intensive screening and investigations. However, one could take the position that all the steps noted above should be part of everyone's plan for their later years whether or not there is the possibility of developing cognitive impairment or not. Since the population and demographic possibility of developing some degree of cognitive impairment over the age of 80 years approaches as much as ~35%, one can argue that such planning is suitable and recommended for everyone.

## 3. If No Specific Pharmacological Treatment Exists, What Does One Do?

 As a consequence of the realization that there are no clear pharmacological interventions, whether with medications or vaccines that appear to be effective, the default recommendations at present are of life-style management. Such recommendations are the result of either population-based or indirect studies from the world of vascular-based diseases (heart and stroke) or on assumptions about the impact of neurological challenge through thought-stimulating efforts and maneuvers. Much of the evidence is indirect or associative. The argument in favor is that since such interventions are essentially safe and may have unexpected benefits such as increased socialization and also provides some element of control (especially to patients in the earlier stages of cognitive impairment or family members or the so-called worried well), some potential beneficial impact over a future that may seem depressingly somber may be deemed worthwhile. Such steps in the lifestyle and brain-stimulation domains are often and almost universally recommended by those who practice in the field of dementia [14–17]. One of the problems in dealing with the “worried well” is that despite reassurance from tests actually undertaken that prove to be “negative,” the impact on the process of worrying is not necessarily allayed [18].

## 4. Tools for Early Diagnosis

 The latest major entry into the field of “early identification” of individuals at risk of dementia is the outcome of studies on a range of cognitive tests that appear to be a fairly good predictor of likely future development of a cognitive impairment/dementia condition that can be administered by nonphysicians and can be used for a variety of purposes. The COGNIGRAM is a simple computerized battery of tests based on a card game. The actual product is based on work published among other places in the Journal of Clinical and Experimental Neuropsychology in 2012 in which the conclusion states that “The aim of this study was to validate the CogState Brief Battery, which assesses psychomotor, attentional, working memory, and visual learning functions, in healthy older people and in patients with mild cognitive impairment (MCI) and Alzheimer's disease (AD), enrolled in the Australian Imaging, Biomarkers and Lifestyle (AIBL) study. These results suggest that the CogState Brief Battery can be used to screen for AD-related cognitive changes.” [19]. There have been other tests over the years that are purportedly useful in determining the cognitive status of individuals and defining those who have evidence of mild cognitive impairment or are at risk. An example of a previous test of this nature is the CAMCOG which has been in existence and used in various studies for many years [20]. The presumed advance of the COGNIGRAM is the computerization, ease of administration, and the studies supporting its efficacy. The issue at present is not whether one neuropsychological battery of tests is more efficacious than other one, but whether any of them should be used as a *screening* for an otherwise apparently health population as opposed to a test used to confirm a clinical diagnosis in someone showing evidence of some cognitive aberrations that require careful review, assessment, and followup.

 As is often the case in the *health care industry,* there are always investors who are interested in health-care related products, especially those that have a huge potential audience of interested parties and potential consumers. To this effect, a study in the financial media dealing with this initiative states that “Cogstate bets big on its cognitive test Cognigram” as reported in the February 13, 2013, edition of BioSpectrum, a biotech-directed on-line publication. In this article it states that “Cogstate is preparing for the imminent commercial launch of its cognitive test to general physicians in Canada, with its partner Merck. The test, branded as Cognigram, allows physicians to identify subtle changes in cognitive function and is being positioned as a tool to help doctors to detect the early stages of cognitive decline associated with a neurodegenerative disease.” “We are very pleased with the progress that Merck is making to roll out Cognigram in Canada and expect to see the first revenues from the launch during this financial year. Merck has invested a significant amount of time and resources in preparing the product and its team for launch and is very committed to the success of Cognigram. Like us, they regard it is a major opportunity”, there were the words of one of the company's spokespeople [21]. One of the issues reflected by this enthusiasm by a respected analyst of the “business” of dementia is the necessity of some effective regulatory basis for the potential innovation in markers for future disease that could lead to meaningful research without causing another level of problems for the individuals who seek or agree to the testing [22].

## 5. What Is the Appropriate Medical Advice?

 When patients come to us asking about this test which can be expected to be heavily marketed, what is the advice that we should give? There has already been a substantial effort to promote interest and participation in “educational sessions” sponsored by Merck for doctors who not only see many elderly individuals but especially those who might act as opinion leaders. The goal would be to promote the utilization of such tests especially among primary care physicians in lieu of the often imprecise and time consuming generally accepted office cognitive screening tests such as Folstein's MMSE (mini-mental state examination) and the MOCA (Montreal Cognitive Assessment) [23, 24] or the aforementioned CAMCOG [20].

 The way the question of such testing is approached can have a huge impact on the physical and emotional health of the individual who completes the examination (for which it is expected that there will be a private fee as it is not expected to be covered by the Canadian provincial, American Medicare, or private health care insurance plans) as well as family members and the health care industry. One can imagine the implications of a *positive* screening test in an otherwise healthy person without subjective or objective symptoms of cognitive problems or ones that have not been deemed significant when it comes to further investigations. One might expect a virtual explosion of imaging and blood screening studies for those conditions which in the past have been suggested as indicators of potentially “reversible” causes of dementia even though that concept has been dispelled in the academic literature—one continues to see “workups” for dementia that include a wide array of laboratory tests even in the face of absence of any symptoms or signs that there might be a reason to expect an abnormality in that particular laboratory domain. An article published by Clarfield in 2003 entitled “The decreasing prevalence of reversible dementias: an updated meta-analysis” found that “Alzheimer's disease was still the commonest cause of dementia (56.3%) followed by a vascular aetiology (20.3%). Conditions requiring neuroimaging made up only 2.2% of cases. Potentially reversible causes were seen in 9%, and only 0.6% of dementia cases actually reversed (0.29% partially, 0.31% fully)” and concluded that “The reported proportion of dementias that reverse is much lower than previously thought. While comorbidity should always be treated for its own sake with the added hope that cognitive decline may at least be delayed, the present findings have significant clinical and economic implications for the workup of dementia” [25].

 If there were agents for which the possibility of altering the course of cognitive decline was deemed through appropriate animal model or other studies, we could anticipate that research protocols might be developed which seek the participation of otherwise healthy individuals or those with subjective concerns about cognition or strong family histories. Such individuals might be willing to undergo the battery of tests to determine their potential risk prior to enrolling in what might be a prolonged study with either pharmacological agents or a combination of pharmacological and life-style modifications to determine potential long-term benefits.

## 6. Recommendations

 In keeping with the ethical principle of nonmaleficence, one would have to demonstrate a very positive countering ethical principle of beneficence in order to justify the *wholesale* introduction of screening tests or batteries of tests to make an early diagnosis of a definable clinical state affecting cognition which is likely to progress to a dementia-like condition. The tests in question include the new computer-based neurocognitive tests such as Cognigram or biological markers or sophisticated imaging procedures, all are appearing at a rapid rate. In the absence of known and verified interventions that have been shown to change the trajectory of the development of the condition, the knowledge of one's increased risk might have more negative (maleficent) effects than positive (beneficent) effects. The potentially negative effects beyond the emotional are related to the impact on such issues as insurance eligibility for what would likely be construed as a “preexisting” condition. This unanticipated effect may not be clearly expressed or understood by those agreeing to such avenues of testing and may reflect local health care systems, insurance structures, and the implications for employment, health care, and personal decision making [7].

 At this point in the development of approaches to avoid whatever it is that results in the development of cognitive impairment/dementia, the focus on vascular risk factor modification and challenges to the brain seem to be the most effective steps for which there are no obvious negative impacts. If anything the steps that are apparently “good for the brain” seemed to be “good for the heart”. In the face of this is the proviso that physicians know that life-style modification is one of the most difficult things for individuals to undertake. We all have experienced the resistance to such steps in the field of cardiovascular disease, even when real risk or events have been experienced. Will a “diagnosis” of “possible” or “impending” dementia improve adoption or adherence to life style changes? Perhaps yes or perhaps no more than a high Framingham risk assessment makes people change their diet and begin exercising. 

 We should on the other hand be alerted and be willing to modify our approach if within the world of dementia research modalities of intervention are deemed of high enough potential that individuals might be willing to risk the potentially negative impact of screening and determining their level of risk for future cognitive decline or dementia. If and when that comes to pass, there may be a move to review again the process of screening for the purposes of defining the highest risk individuals for focused interventions or well-designed research protocols.

Doctors and those in the world of public health, population health, health promotion, and advertising should be putting their heads together to see what can be done to invoke the risk of cognitive decline and dementia as a reason to embrace those positive and beneficial life-style changes that appear to be promising. That should be possible within the current understanding of evidence to postpone or prevent what is considered by most as the most devastating disease and one that has enormous implications for individuals, their families, our society, and the integrity of the current and future health care system. 

Before we collectively fall into a potential “sinkhole” of health care resource utilization because of people worried about their cognitive future, we have to consider the implications carefully. At this time, with little therapeutically to offer other than basic and inexpensive life-style modifications and mental and social stimulation strategies, we must demand proper controlled studies to determine whether or not “early identification” with subsequent specific and beneficial pharmacological interventions of one sort or another will result in some tangible and measurable benefit, clinically, psycho-socially, and economically.
